# Microarray-based analysis and clinical validation identify ubiquitin-conjugating enzyme E2E1 (*UBE2E1*) as a prognostic factor in acute myeloid leukemia

**DOI:** 10.1186/s13045-016-0356-0

**Published:** 2016-11-17

**Authors:** Hongmei Luo, Yu Qin, Frederic Reu, Sujuan Ye, Yang Dai, Jingcao Huang, Fangfang Wang, Dan Zhang, Ling Pan, Huanling Zhu, Yu Wu, Ting Niu, Zhijian Xiao, Yuhuan Zheng, Ting Liu

**Affiliations:** 1 Department of Hematology, Hematology Research Laboratory, West China Hospital, Sichuan University, #37 GuoXue Xiang Street, Chengdu, 610041 China; 2 Department of Internal Medicine, Weiss Memorial Hospital, University of Illinois, Chicago, IL USA; 3 Department of Haemotologic Oncology, Taussig Cancer Center, Cleveland Clinic, Cleveland, OH USA; 4 Sinopec Southwest Company, Chengdu, China; 5 State Key Laboratory of Biotherapy and Cancer Center, Sichuan University, Chengdu, China; 6 Chinese Academy of Medical Sciences, Institute of Hematology and Blood Diseases Hospital, Tianjin, China

**Keywords:** Acute myeloid leukemia, UBE2E1, Prognosis

## Abstract

**Background:**

Previous research suggested that single gene expression might be correlated with acute myeloid leukemia (AML) survival. Therefore, we conducted a systematical analysis for AML prognostic gene expressions.

**Methods:**

We performed a microarray-based analysis for correlations between gene expression and adult AML overall survival (OS) using datasets GSE12417 and GSE8970. Positive findings were validated in an independent cohort of 50 newly diagnosed, non-acute promyelocytic leukemia (APL) AML patients by quantitative RT-PCR and survival analysis.

**Results:**

Microarray-based analysis suggested that expression of eight genes was each associated with 1-year and 3-year AML OS in both GSE12417 and GSE8970 datasets (*p* < 0.05). Next, we validated our findings in an independent cohort of AML samples collected in our hospital. We found that ubiquitin-conjugating enzyme E2E1 (*UBE2E1*) expression was adversely correlated with AML survival (*p* = 0.04). Multivariable analysis showed that *UBE2E1* ^high^ patients had a significant shorter OS and shorter progression-free survival after adjusting other known prognostic factors (*p* = 0.03). At last, we found that *UBE2E1* expression was negatively correlated with patients’ response to induction chemotherapy (*p* < 0.05).

**Conclusions:**

In summary, we demonstrated that *UBE2E1* expression was a novel prognostic factor in adult, non-APL AML patients.

**Electronic supplementary material:**

The online version of this article (doi:10.1186/s13045-016-0356-0) contains supplementary material, which is available to authorized users.

## Background

Acute myeloid leukemia (AML), characterized by expansion of malignant myeloid precursor cells in peripheral blood and bone marrow, is the most prevalent acute leukemia in adults [1]. Several AML prognostic factors have been reported, including patient age and cytogenetic features [2, 3]. Interestingly, Metzeler et al showed that high expression of lymphoid enhancer binding factor-1 (*LEF1*) is a favorable AML prognostic factor in non-acute promyelocytic leukemia (APL) AML [4]. This study provided insights on prognostic single gene expression in AML. Therefore, we performed a systematical microarray-based analysis to search gene expression that correlates with AML overall survival (OS).

## Methods

### Microarray datasets download and analysis

We selected AML microarray datasets from Oncomine (www.oncomine.com). Our selection criteria included (i) microarray examining adult AML patient samples; (ii) array data and patient survival data were both published; (iii) microarray data quality; and (iv) microarray used affymetrix array platform. Based on those selection criteria, we used GSE12417 and GSE8970 datasets for our analysis [5, 6]. GSE12417 had 2 independent cohort of samples, which were examined by affymetrix platforms GPL570 and GPL96, respectively. Specifically, GSE12417-GPL96 dataset included 163 adult AML patient gene expression profiles, while GSE12417-GPL570 dataset included 79 adult AML patient gene expression profiles. The patients were previously untreated and received cytarabine-based intensive induction and consolidation chemotherapy in the trial [4, 5]. GSE8970 dataset used affymetrix platform GPL96. GSE8970-GPL96 dataset included 34 adult AML patient samples. The patients were pretreated with tipifarnib [6]. The stem cell transplantation status of those patients was not available. The same probe ID system was used in all above datasets, enabling results to be cross-compared. Gene expression profiles of above datasets were downloaded from NCBI Gene Expression Omnibus database. Clinic information of those patients was downloaded from Oncomine.

**Fig. 1:**
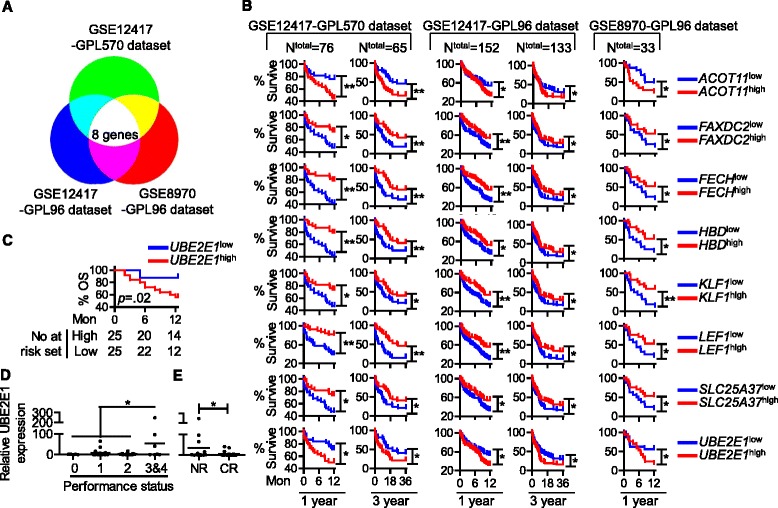
**a** Diagram showing the principal of microarray-based analysis. **b** OS according to high gene expression (gene^high^, *red*) and low gene expression (gene^low^, *blue*), analyzed using microarray datasets GSE12417 and GSE8970. GSE12417 had two independent cohorts, examined by two microarray plateforms GPL570 and GPL96. **c** OS according to *UBE2E1* ^high^ (*red*) and *UBE2E1* ^low^ (*blue*) in validation cohort. **d** Relative *UBE2E1* expression in patients with different performance status. **e** Relative *UBE2E1* expression in patients with different responses to induction chemotherapy, no response (*NR*) vs. complete response (*CR*). (*p < 0.05, **p < 0.01)

Our algorithm of prognostic genes identification was to identify prognostic genes in each microarray dataset and then find common prognostic genes across all tested datasets to avoid bias associated with single microarray dataset. In one dataset, single gene expression in each AML patient sample was presented by probe intensity. Patients with a probe intensity value above or below the median of all samples were categorized in probe^high^ and probe^low^ groups, respectively. Survival (1 year and 3 years) of the two groups was compared by the Mantel-Cox test, and *p* < 0.05 was considered significant. Such calculation was repeated for all genes (probes) in the dataset by programming in R software to generate a list of prognostic genes. Common genes across both datasets were identified using the same probe ID (Fig. 1a).

### Patient samples

Our validation cohort had 50 newly diagnosed AML patient samples collected at West China Hospital, Sichuan University from 2010 to 2011. The inclusive criteria include (1) adult patients (age > 18); (2) patients with newly diagnosed AML except non-APL subtype; and (4) no chemotherapy was administered before the study. Bone marrow cell samples of the patients were collected as described previously [7]. All patients were treated based on the standard protocol including anthracyclines plus cytarabine. The study was reviewed and approved by the Central Ethics Committees of Institute of Hematology/Blood Diseases Hospital, Chinese Academy of Medical Sciences, and was filed in and permitted by the Ethics Committees of West China Hospital, Sichuan University.

### Quantitative RT-PCR

Total RNA was extracted from patient bone marrow cells with RNeasy Mini Kit (QIAGEN) according to the manufacturer’s instruction. The expression of target genes was analyzed by qPCR using SYBR green real-time PCR system (Bio-Rad). The expression of housekeeping gene GAPDH was used as an internal control. Primers used were described in Table 1.

**Table 1:** Probes for quantitative RT-PCR

Target gene	Primers	Sequence
*GAPDH*	Forward	GTCTCCTCTGACTTCAACAGCG
	Reverse	ACCACCCTGTTGCTGTAGCCAA
*LEF1*	Forward	TGCCAAATATGAATAACGACCCA
	Reverse	GAGAAAAGTGCTCGTCACTGT
*FECH*	Forward	GGAGATGTTCACGACTTCCTTC
	Reverse	GAATGGTGCCAGCTTATTCTGA
*HBD*	Forward	GAATGGTGCCAGCTTATTCTGA
	Reverse	ACACCAGCCACCACCTTCTGAT
*ACOT11*	Forward	CATCGTGAACAATGCCTTCAAAC
	Reverse	GTCCAGGACCACAAAGGTCAT
*KLF1*	Forward	GGTTGCGGCAAGAGCTACA
	Reverse	GTCAGAGCGCGAAAAAGCAC
*FAXDC2*	Forward	ATTGGTGGTTGACACAACAGG
	Reverse	AGAACTGTGCGGATAGACTGG
*SLC25A37*	Forward	AGAAAATCATGCGGACCGAAG
	Reverse	TGGTGGTGGAAAACGTCATTTA
*UBE2E1*	Forward	CCTCCAAAGGTTACATTTCGGA
	Reverse	GGTCGGCAGGATTACAGTCTG

### Statistical analysis

Patients’ characteristics between *UBE2E1* ^high^ and *UBE2E1* ^low^ groups were analyzed using Fisher’s exact test. The association between *UBE2E1* expression as well as other prognostic factors and patients’ survival was investigated using univariable Cox regression and multivariable logistic regression analysis. All above statistical analyses were performed in SPSS version 22 software. Patient survival was graphed and analyzed using GraphPad Prism 5 software with Mantel-Cox test (a function of GraphPad Prism 5). A *p* < 0.05 was considered statistically significant.

## Results

### Microarray-based analysis for AML prognostic gene expression

The microarray-based analysis showed that eight probes’ (genes’) expression was each associated with AML OS in all datasets, from 1-year survival to 3-year OS (Fig. 1b). These genes were *ACOT11*, *FAXDC2*, *FECH*, *HBD*, *KLF1*, *LEF1*, *SLC25A37*, and *UBE2E1* (Table 2 for chi-square and *p* value). As shown in Fig. 1b, the expression of *FAXDC2*, *FECH*, *HBD*, *KLF1*, *LEF1*, and *SLC25A37* was a favorable prognostic factor for AML, while high expression of *UBE2E1* and *ACOT11* was associated with poor OS (*p* < 0.05). Furthermore, we compared the target genes’ expression in normal BM vs. AML BM. At least in two tested microarray datasets GSE13159 and GSE1159, all target genes were aberrantly expressed in AML: AML patients had averagely increased *ACOT11* and *UBE2E1* gene expression, while the patients had lower expression of the other genes (Additional file 1: Figure S1). In addition, we conducted multivariable analyses of the microarray datasets. The results revealed that only *UBE2E1*, *LEF1,* and *FECH1* were independent prognostic factors in AML, despite the impact of the patient age, FAB subtype as well as other prognostic factors (Table 3). Interestingly, among those three identified prognostic-related single genes, high expression of *LEF1* has already been reported as a favorable prognostic factor in cytogenetically normal adult AML [4].

**Table 2:** Microarray-based analysis for AML overall survival related gene expression

		GSE12417-GPL570				GSE12417-GPL96				GSE8970-GPL96	
		1-year		3-year		1-year		3-year		1-year	
Gene name	Probe ID	Chi-square	*p* value	Chi-square	*p* value	Chi-square	*p* value	Chi-square	*p* value	Chi-square	*p* value
*ACOT11*	214763_at	6.941523	0.00842	11.8655	0.00057	3.88562	0.0487	4.72131	0.02979	4.03782	0.04449
*KLF1*	210504_at	5.748149	0.01651	4.25163	0.03921	4.10844	0.04267	9.61817	0.00193	5.14706	0.02329
*LEF1*	221558_s_at	14.72791	0.00012	13.5199	0.00024	7.77776	0.00529	11.0276	0.0009	5.14706	0.02329
*FECH*	203115_at	11.52174	0.00069	9.90671	0.00165	7.00522	0.00813	10.0492	0.00152	5.14706	0.02329
*FAXDC2*	220751_s_at	5.786984	0.01615	7.59189	0.00586	4.01214	0.04517	7.41339	0.00647	5.14706	0.02329
*HBD*	206834_at	11.87311	0.00057	7.12887	0.00759	4.42625	0.03539	6.57289	0.01035	5.14706	0.02329
*SLC25A37*	221920_s_at	5.786984	0.01615	5.01703	0.0251	4.59061	0.03215	6.62943	0.01003	5.14706	0.02329
*UBE2E1*	212519_at	4.114544	0.04252	4.50706	0.03376	6.25573	0.01238	5.73031	0.01667	4.03782	0.04449

**Table 3:** Multivariable analysis of target genes in microarray datasets

		GSE8970-GPL96				GSE12417-GPL96		GSE12417-GPL570	
		OS		PFS		OS		OS	
		HR (95% CI)	*p*	HR (95% CI)	*p*	HR (95% CI)	*p*	HR (95% CI)	*p*
*ACOT11*	*ACOT11* expression, high vs. low	0.658(0.296,1.462)	0.304	0.636(0.287,1.405)	0.263	1.445(0.970,2.154)	0.07	1.809(1.009,3.243)	0.047
	Age, per 10-year increase	1.201(0.704,2.048)	0.501	1.212(0.716,2.053)	0.474	1.295(1.126,1.490)	<0.001	1.400(1.088,1.800)	0.009
	Sex, male vs. female	1.326(0.473,3.717)	0.592	1.369(0.488,3.838)	0.551	NA	NA	NA	NA
	Prior myelodysplastic syndrome	0.685(0.270,1.741)	0.427	0.711(0.281,1.795)	0.470	NA	NA	NA	NA
	Organ dysfunction	1.162(0.490,2.753)	0.733	1.253(0.536,2.930)	0.603	NA	NA	NA	NA
	FAB subtype	NA	NA	NA	NA	0.879(0.773,0.999)	0.048	0.956(0.778,1.174)	0.667
*FAXDC2*	*FAXDC2* expression, high vs. low	0.922(0.420.2.023)	0.839	1(0.462,2.166)	>0.99	0.869(0.590,1.280)	0.477	0.791(0.444,1.406)	0.424
	Age, per 10-year increase	1.185(0.685,2.051)	0.544	1.182(0.688,2.030)	0.545	1.278(1.114,1.466)	<0.001	1.382(1.074,1.778)	0.012
	Sex, male vs. female	1.553(0.589,4.094)	0.373	1.662(0.636,4.339)	0.300	NA	NA	NA	NA
	Prior myelodysplastic syndrome	0.681(0.265,1.753)	0.426	0.698(0.273,1.783)	0.452	NA	NA	NA	NA
	Organ dysfunction	1.094(0.471,2.539)	0.835	1.164(0.509,2.662)	0.719	NA	NA	NA	NA
	FAB subtype	NA	NA	NA	NA	0.862(0.762,0.976)	0.019	0.965(0.782,1.192)	0.744
*FECH*	*FECH* expression, high vs. low	0.363(0.149,0.883)	0.025	0.390(0.163,0.934)	0.034	0.566(0.382,0.838)	0.005	0.424(0.235,0.764)	0.004
	Age, per 10-year increase	1.050(0.625,1.762)	0.854	1.066(0.639,1.779)	0.806	1.277(1.109,1.470)	0.001	1.410(1.095,1.817)	0.008
	Sex, male vs. female	1.932(0.734,5.089)	0.183	1.988(0.763,5.181)	0.160	NA	NA	NA	NA
	Prior myelodysplastic syndrome	0.452(0.169,1.204)	0.112	0.486(0.184,1.284)	0.146	NA	NA	NA	NA
	Organ dysfunction	1.684(0.653,4.343)	0.281	1.781(0.697,4.554)	0.228	NA	NA	NA	NA
	FAB subtype	NA	NA	NA	NA	0.855(0.757,0.967)	0.013	0.978(0.790,1.210)	0.837
*HBD*	*HBD* expression, high vs. low	0.396(0.166,0.942)	0.036	0.430(0.183,1.011)	0.053	0.528(0.356,0.783)	0.001	0.479(0.266,0.862)	0.014
	Age, per 10-year increase	1.015(0.594,1.732)	0.957	1.035(0.610,1.756)	0.898	1.294(1.126,1.487)	<0.001	1.363(1.070,1.738)	0.012
	Sex, male vs. female	2.086(0.738,5.898)	0.166	2.125(0.765,5.905)	0.148	NA	NA	NA	NA
	Prior myelodysplastic syndrome	0.595(0.221,1.605)	0.305	0.627(0.236,1.666)	0.349	NA	NA	NA	NA
	Organ dysfunction	1.479(0.595,3.681)	0.400	1.576(0.637,3.895)	0.325	NA	NA	NA	NA
	FAB subtype	NA	NA	NA	NA	0.861(0.763,0.973)	0.016	0.995(0.805,1.229)	0.96
*KLF1*	*KLF*1 expression, high vs. low	0.457(0.208,1.000)	0.050	0.429(0.198,0.927)	0.031	0.560(0.379,0.829)	0.004	0.581(0.325,1.037)	0.066
	Age, per 10-year increase	1.061(0.609,1.849)	0.834	1.062(0.613,1.840)	0.830	1.281(1.111,1.476)	0.001	1.382(1.087,1.756)	0.008
	Sex, male vs. female	1.928(0.693,5.362)	0.209	2.064(0.742,5.738)	0.165	NA	NA	NA	NA
	Prior myelodysplastic syndrome	0.562(0.204,1.547)	0.265	0.574(0.208,1.584)	0.284	NA	NA	NA	NA
	Organ dysfunction	1.184(0.492,2.845)	0.706	1.259(0.530,2.994)	0.602	NA	NA	NA	NA
	FAB subtype	NA	NA	NA	NA	0.853(0.755,0.963)	0.011	0.976(0.791,1.204)	0.821
*LEF1*	*LEF1* expression, high vs. low	0.273(0.117,0.638)	0.003	0.287(0.126,0.655)	0.003	0.605(0.407,0.901)	0.013	0.382(0.209,0.700)	0.002
	Age, per 10-year increase	1.328(0.789,2.236)	0.285	1.342(0.802,2.245)	0.263	1.259(1.094,1.449)	0.001	1.423(1.099,1.843)	0.007
	Sex, male vs. female	2.475(0.793,7.723)	0.119	2.511(0.821,7.673)	0.106	NA	NA	NA	NA
	Prior myelodysplastic syndrome	0.554(0.208,1.479)	0.239	0.595(0.225,1.573)	0.295	NA	NA	NA	NA
	Organ dysfunction	1.021(0.406,2.586)	0.966	1.109(0.450,2.733)	0.823	NA	NA	NA	NA
	FAB subtype	NA	NA	NA	NA	0.870(0.766,0.988)	0.031	1.031(0.829,1.282)	0.785
*SLC25A37*	*SLC25A3*7 expression, high vs. low	0.312(0.117,0.827)	0.019	0.294(0.111,0.782)	0.014	0.540(0.360,0.808)	0.003	0.616(0.345,1.101)	0.102
	Age, per 10-year increase	0.963(0.554,1.674)	0.894	0.961(0.557,1.661)	0.888	1.292(1.122,1.488)	<0.001	1.389(1.084,1.778)	0.009
	Sex, male vs. female	1.431(0.548,3.737)	0.465	1.474(0.568,3.828)	0.425	NA	NA	NA	NA
	Prior myelodysplastic syndrome	0.336(0.106,1.065)	0.064	0.332(0.105,1.058)	0.062	NA	NA	NA	NA
	Organ dysfunction	1.377(0.573,3.310)	0.475	1.494(0.629,3.552)	0.363	NA	NA	NA	NA
	FAB subtype	NA	NA	NA	NA	0.897(0.792,1.015)	0.086	0.983(0.796,1.214)	0.872
*UBE2E1*	*UBE2E1* expression, high vs. low	3.5(1.08,11.33)	0.04	3.9(1.27,11.98)	0.02	1.28(0.77,2.12)	0.04	2.02(1.8,3.79)	0.03
	Age, per 10-year increase	1.04(0.96,1.13)	0.32	1.04(0.96,1.12)	0.35	1.02(1.00,1.04)	0.03	1.36(1.07,1.73)	0.01
	Sex, male vs. female	1.22(0.39,3.81)	0.74	1.51(0.52,4.42)	0.45	NA	NA	NA	NA
	Prior myelodysplastic syndrome	0.83(0.25,2.72)	0.75	0.88(0.29,2.71)	0.83	NA	NA	NA	NA
	Organ dysfunction	0.96(0.33,2.81)	0.94	0.99(0.37,2.6)	0.98	NA	NA	NA	NA
	FAB subtype	NA	NA	NA	NA	0.87(0.74,1.0)	0.1	1.06(0.85,1.32)	0.61

*NA* not available

### High expression of UBE2E1 is a poor prognostic factor in AML

We validated our findings in an independent cohort of 50 AML patients (median age 43). Target gene expression was analyzed by quantitative RT-PCR. Based on median gene expression, we divided our patients into two study groups, gene^high^ and gene^low^ groups. The survival analysis showed that out of eight genes identified by microarray studies, the expression of only one gene *UBE2E1* (ubuquitin-conjugating enzyme E2E1) was associated with AML OS in our validation cohort, and this gene was one of the three genes with independent prognostic value on multivariable analysis in the training set. The *UBE2E1* ^high^ group had a markedly shorter OS compared with *UBE2E1* ^low^ group (*p* = 0.02; Fig. 1c). Expression of the other seven genes was not associated with AML prognosis in our study (*p* > 0.05; Additional file 1: Figure S2). We could not detect *KLF1* expression in AML patient samples, although the qPCR primers for this gene were validated.

Next, we performed multivariable analysis to verify the prognostic significance of *UBE2E1* expression in our validation cohort. The patient characteristics of *UBE2E1* ^high^ and *UBE2E1* ^low^ groups are shown in Table 4. No significant difference in patient characteristics, such as age, FAB subtypes, WBC count, BM blast percentages, gene mutations, was found between the two groups. We found no difference in patients’ treatment between those two groups (Table 5). *UBE2E1* ^high^ patients had a short OS (*p* = 0.04) as well as a short progression-free survival (*p* = 0.03) compared with *UBE2E1* ^low^ patients after adjusting for the impact of other prognostic factors including patient age, gender, performance status, and response to induction chemotherapy (Table 6).

**Table 4:** Characteristic of AML patients in validation cohort

Variable	*UBE2E1* ^high^ *n* = 25	*UBE2E1* ^low^ *n* = 25	*p* value
Median age	43.08	43.25	0.567
Female, no. (%)	10(40)	13(52)	0.395
Secondary or treatment-related AML, no. (%)	2(8)	2(8)	>0.99
FAB subtype , no.			0.838
M_1_	3	3	
M_2_	10	12	
M_4_	7	5	
M_5_	1	3	
M_6_	2	0	
NA	2	2	
Median WBC, 10^9^/L (range)	6.83(0.3–244.38)	23.88(0.68–366)	0.289
Median BM plasts, %, (range)	49.5(11–94)	65.5(22–90.5)	0.175
Median platelet count, 10^9^/L (range)	39(4–151)	35.5(4–180)	0.918
*CEBPA* mutated, no. (%)	2(8)	5(20)	0.179
Missing date	1	3	
*NPM1* mutated, no. (%)	2(8)	4(16)	0.327
Missing date	1	3	
*IDH1* mutated, no. (%)	0	0	>0.99
Missing date	1	3	
*FLT3*-*TKD* mutated, no. (%)	0	1(4)	0.296
Missing date	1	3	
*FLT3*-*ITD* mutated, no. (%)	0	2(4)	0.149
Missing date	1	3	
*AML1*/*ETO* mutated, no. (%)	4(16)	6(24)	0.389
Missing date	1	3	
*C-KIT* D816V mutated, no. (%)	1(4)	2(8)	0.504
Missing date	1	3	
*CBEB*-*MYH11* mutated, no. (%)	2(8)	3(12)	0.568
Missing date	1	3

**Table 5:** Characteristic of AML patients treatment in validation cohort

Variable	*UBE2E1* ^high^	*UBE2E1* ^low^	*p*
Treatment, no.(%)	25(100)	25(100)	0.422
CAG	2(8)	1(4)	
DA	9(36)	11(44)	
QA	1(4)	0	
HAD	1(4)	0	
IDA	6(24)	11(44)	
D-CAG	2(8)	1(4)	
Allo-HSCT	0(0)	0(0)

**Table 6:** Multivariable analysis in validation cohort

	OS		PFS	
	HR (95% CI)	*p*	HR (95% CI)	*p*
*UBE2E1* expression, high vs. low	3.227(1.05,9.852)	0.040	3.818(1.616,12.553)	0.027
Age, per 10-year increase	1.666(1.112,2.498)	0.013	1.536(1.02,2.313)	0.040
Sex, male vs. female	0.628(0.214,1.84)	0.396	0.559(0.183,1.71)	0.308
Performance status	0.727(0.374,1.41)	0.345	0.683(0.344,1.358)	0.277
Induction chemo-response	1.472(0.845,2.566)	0.172	1.51(0.853,2.672)	0.157

### UBE2E1 expression and its association with chemotherapy response

Finally, in our validation cohort, low *UBE2E1* expression was associated with a better performance status in the patients (Fig. 1d; *p* < 0.05). We also found that *UBE2E1* expression was associated with response to induction chemotherapy. Patients who had relatively higher *UBE2E1* expression were more likely to achieve no response (NR) to chemotherapy while patients who had lower *UBE2E1* expression were more likely to enter complete remission (CR) (Fig. 1e; *p* < 0.05). This result suggests that *UBE2E1* expression may be a possible predictor for chemotherapy response in AML patients.

## Discussion

In this study, we performed a genome-wide screening to identify gene expression that correlate with adult AML OS. The gene expression profiles (GEPs) from 2 independent datasets of patient samples were used in our analysis. The correlation of each gene expression and AML OS was calculated by a program coded by R software. Only gene identified with statistical significance in both datasets was considered as positive results for further test. By this strategy of analysis, we identified 8 AML prognostic genes. Next, we tested our findings using an independent cohort of 50 AML samples. Our result suggested that although several genes, such as *HBD* and *ACOT11*, had trend correlation, only one gene, *UBE2E1*, was statistically correlated with AML OS in our validation cohort. The negative findings of other 7 genes in our validation cohort might be caused by the relatively small number of patients. In addition, we noticed that the patients in microarray-testing cohort and our validation cohort had different ethnic backgrounds. Further studies might be necessary to draw a more confirmative conclusion.

Mounting evidence has shown that AML is highly heterogeneous and dynamic [8]. The heterogeneous entity of AML emerges from the disease genetic basis, leukemogenesis, pathophysiology, and prognosis. However, cluster of gene expression signature [5], or even single gene expression [4], has been shown to correlate with AML prognosis. Therefore, what is the interpretation of prognostic single gene expression, such as *UBE2E1* and *LEF1*, in AML? We hypothesized that different subgroups of AML, with discrete driver mutations, might have similar epigenetic effectors’ upregulation/downregulation, which correlate with patient’s survival. We also hypothesized that in different prognosis-relevant AML subgroups, the effectors have patterned expression. To test these hypotheses, we performed another microarray-based analysis for *UBE2E1* expression in AML with complex karyotype vs. normal karyotype, *FLT3* mutation vs. wildtype*FLT3*, and *NPM1* mutation vs. wildtype*NPM1*. We selected those genetic abnormalities because they have high frequency of occurrence in AML and correlate with the patient clinical outcome: patients with complex karyotype or *FLT3* mutation had poor treatment outcome, while patients with *NPM1* mutation had good treatment outcome [8]. As shown in Additional file 1: Table S1, complex karyotype or *FLT3*-mutated AML had relatively high *UBE2E1* expression, compared with normal karyotype or wildtype *FLT3* AML, respectively. *NPM1*-mutated AML had relatively low *UBE2E1* expression. These preliminary findings might indicate that *UBE2E1* have patterned expressions, which was well matched with AML classification despite of different genetic basis.

Protein ubiquitination was accomplished by sequential action of enzymes E1, E2, and E3. Specifically, E2 transferred E1-activated ubiquitin to E3, an ubiquitin ligase, and formed an isopeptide bond between ubiquitin and protein substrate. *UBE2E1* was a member of ubiquitin-conjugating enzyme E2 class. Zhu et al. showed that *UBE2E1* regulated *HOX* gene expression by ubiquitinating histones [9]. Thus, *UBE2E1* might play a regulatory role in cell by selectively ubiquitinating target proteins. The function of *UBE2E1* in cell signaling is still largely unknown. However, the regulation of *UBE2E1* on *HOX* gene might be a key to understand the prognostic role of *UBE2E1* in AML. *HOX* gene is a family of highly conserved homeodomain transcription factor genes [10]. There are 39 *HOX* genes, belonging to 4 gene clusters, in human. Previous work has shown that *HOX* genes are aberrantly expressed in AML [11, 12]. Animal study indicated that overexpression *HOX* gene, *HOXA10* and *HOXA9*, promoted AML leukemogenesis [13, 14]. To identify potential *UBE2E1* downstream *HOX* genes, we started with microarray datasets. We found co-expression of *UBE2E1* with *HOXA11* in AML. We also examined *HOXA11* and *UBE2E1* co-expression in our validation cohort (Additional file 1: Figure S3). Interestingly, a recent publication suggested that *HOXA11* expression correlated with glioblastoma patient treatment responses and prognosis [15]. Thus, it is highly possible that *UBE2E1* regulates AML chemoresistance through *HOXA11*.

In our study, we found co-expression of *UBE2E1* with *HOX* family gene, *HOXA11*, in AML. Therefore, we hypothesized that *UBE2E1* regulates *HOXA11* gene expression in AML, and *HOXA11* transcription factor level might be relevant to AML treatment resistance. We are actively conducting more mechanistic studies to demonstrate the role of *UBE2E1* in AML.

## Conclusions

In conclusion, we performed a genome-wide, microarray-based analysis for gene expressions that correlated with AML survival, and found 8 candidate genes. We further tested these genes in an independent validation cohort of 50 AML samples, and identified that *UBE2E1* expression adversely correlated with AML prognosis.

## Additional file

Additional file 1: Figure S1. Aberrant target gene expression in AML. AML patient samples microarray datasets GSE13159 and GSE1159 were downloaded from NCBI. Normal samples in those datasets were bone marrow cells or peripheral blood mononuclear cells (PBMCs) from healthy donors. Target gene expression in normal samples vs. AML patient samples were plotted and compared by the Student’s *t* test (***p* < 0.01). **Figure S2.** Target gene correlation with patient survival in validation cohort. **Figure S3.** Co-expression of UBE2E1 and HOXA11 in AML. **Table S1.** UBE2E1 expression in AML subgroups. (PPTX 202 kb)

## Abbreviations

AML: Acute myeloid leukemia
CR: Complete remission
GEP: Gene expression profile
NR: No response
OS: Overall survival
PFS: Progression-free survival.
