# Supplementary material for: Microarray-based analysis and clinical validation identify ubiquitin-conjugating enzyme E2E1 (UBE2E1) as a prognostic factor in acute myeloid leukemia
Source: J Hematol Oncol. 2016 Nov 17;9:125. doi: 10.1186/s13045-016-0356-0 (PMC5114814; doi:10.1186/s13045-016-0356-0)
Supplement: Additional file 1: Figure S1. — Aberrant target gene expression in AML. AML patient samples microarray datasets GSE13159 and GSE1159 were downloaded from NCBI. Normal samples in those datasets were bone marrow cells or peripheral blood mononuclear cells (PBMCs) from healthy donors. Target gene expression in normal samples vs. AML patient samples were plotted and compared by the Student’s t test (**p < 0.01). Figure S2. Target gene correlation with patient survival in validation cohort. Figure S3. Co-expression of UBE2E1 and HOXA11 in AML. Table S1. UBE2E1 expression in AML subgroups. (PPTX 202 kb) [file 13045_2016_356_MOESM1_ESM.pptx]

## Slide 1
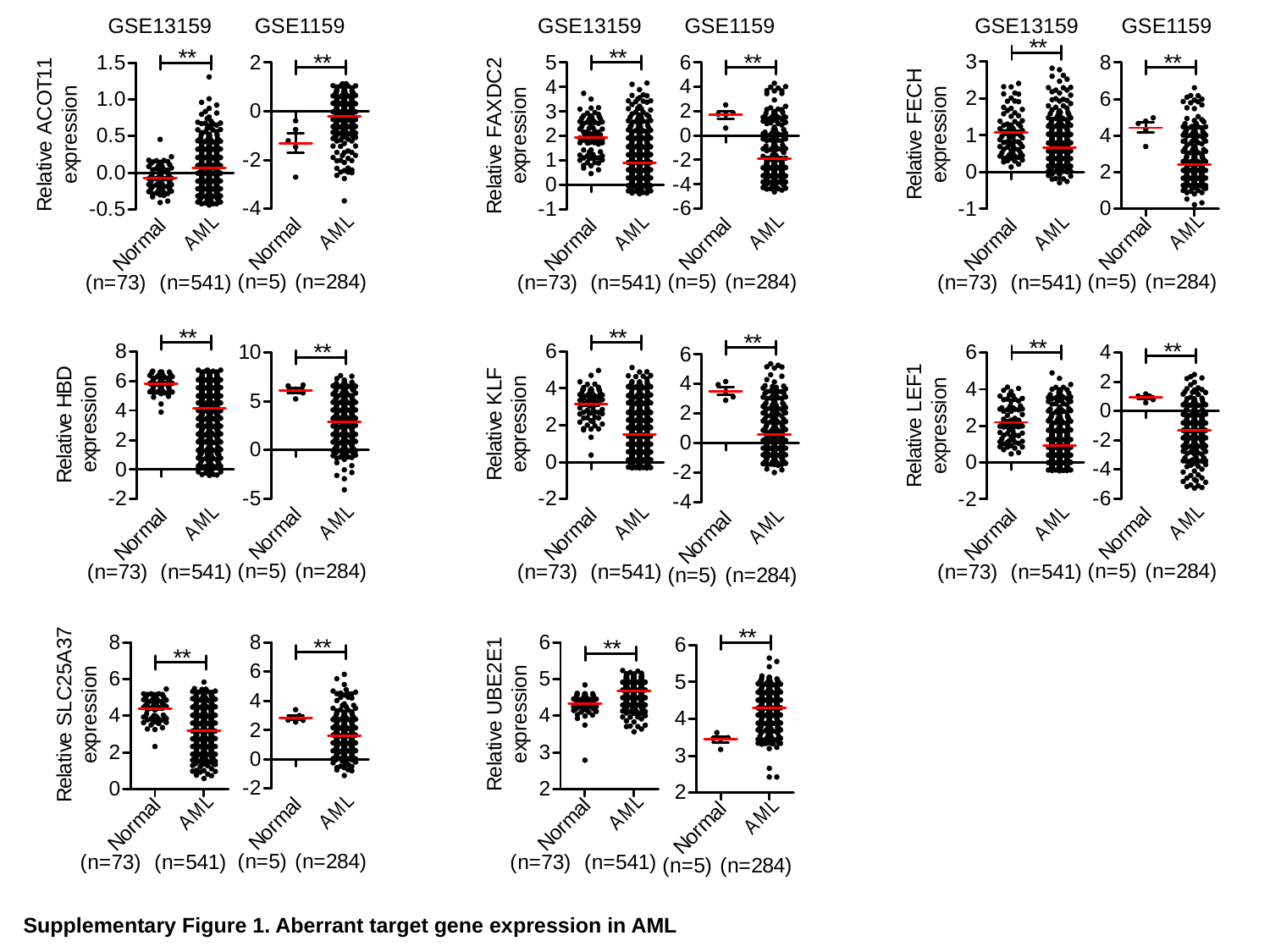

GSE13159
GSE1159
GSE13159
GSE1159
GSE13159
GSE1159
Supplementary Figure 1. Aberrant target gene expression in AML

## Slide 2
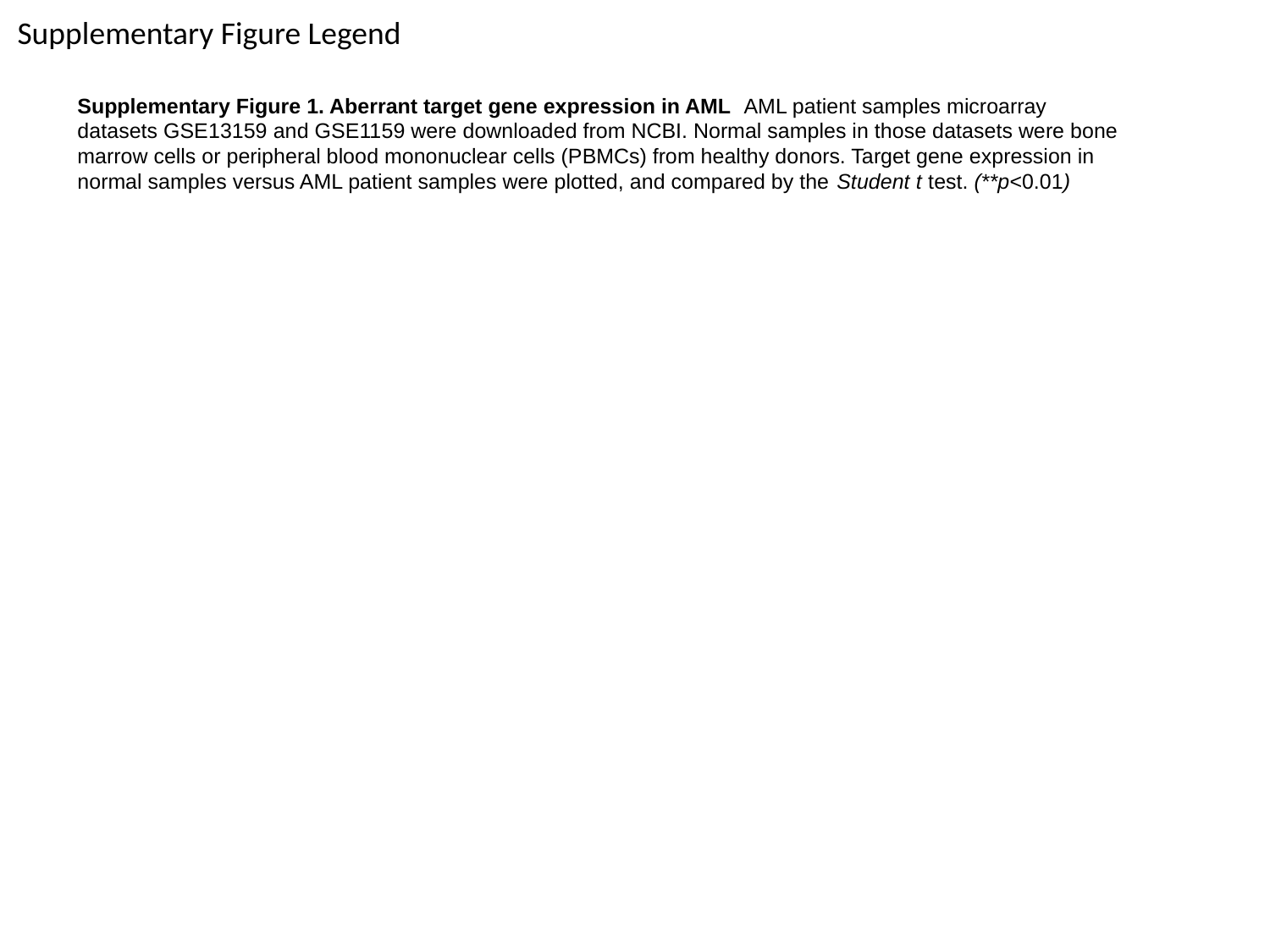

Supplementary Figure Legend
Supplementary Figure 1. Aberrant target gene expression in AML AML patient samples microarray datasets GSE13159 and GSE1159 were downloaded from NCBI. Normal samples in those datasets were bone marrow cells or peripheral blood mononuclear cells (PBMCs) from healthy donors. Target gene expression in normal samples versus AML patient samples were plotted, and compared by the Student t test. (**p<0.01)

## Slide 3
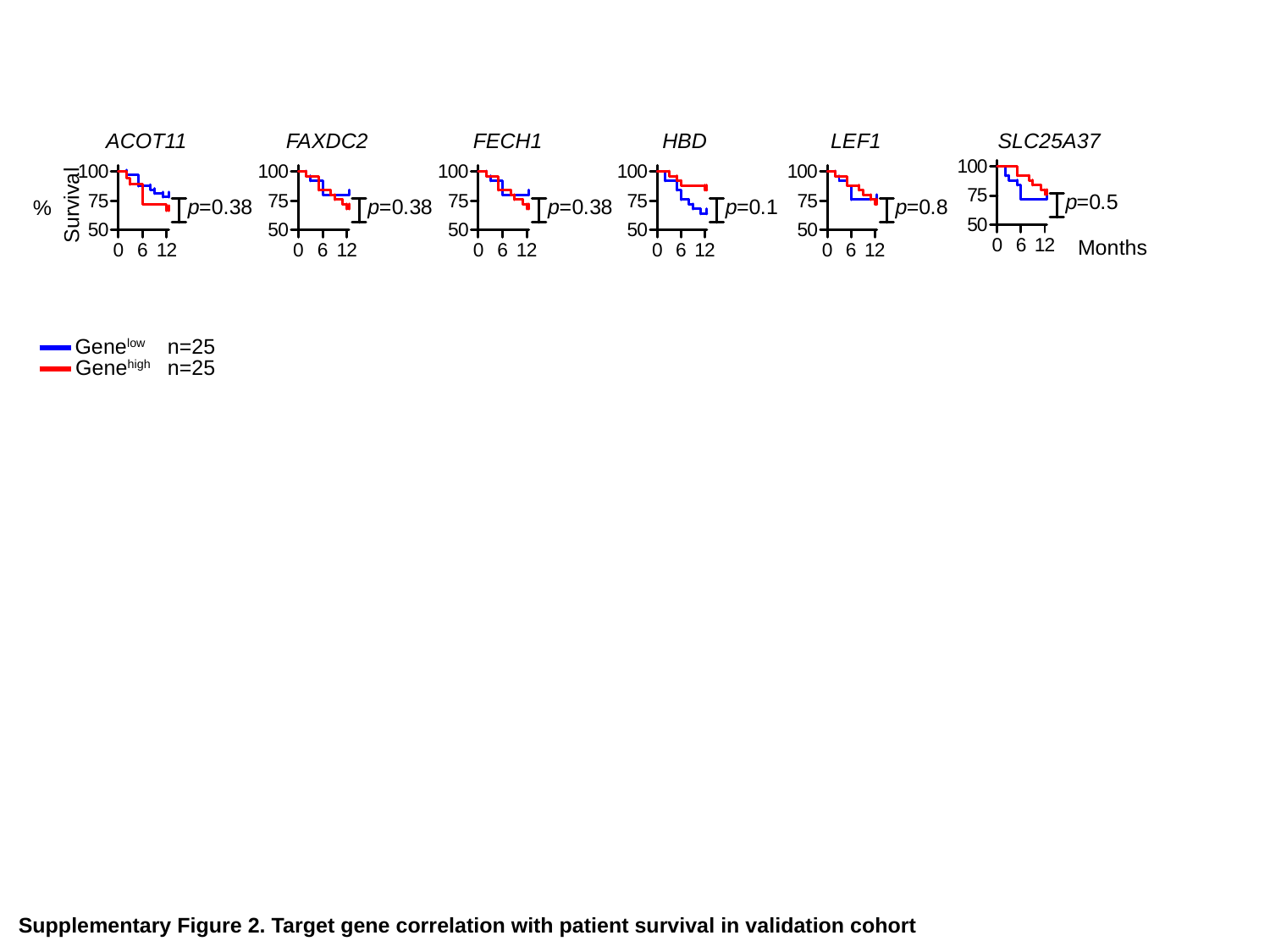

ACOT11
FAXDC2
FECH1
HBD
LEF1
SLC25A37
Survival
%
Months
Genelow
n=25
Genehigh
n=25
Supplementary Figure 2. Target gene correlation with patient survival in validation cohort

## Slide 4
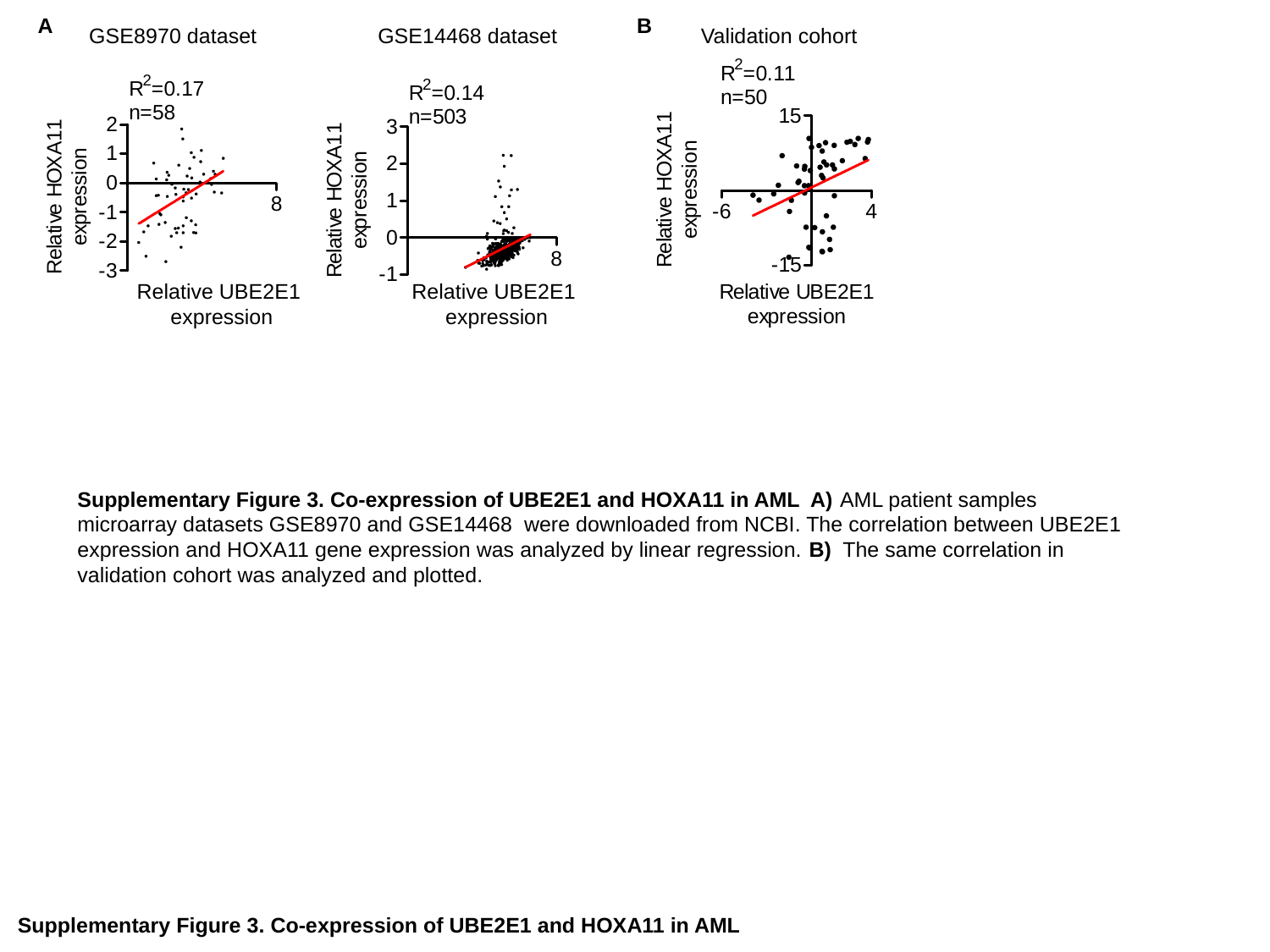

A
B
GSE8970 dataset
Relative UBE2E1
expression
GSE14468 dataset
Relative UBE2E1
expression
Validation cohort
Supplementary Figure 3. Co-expression of UBE2E1 and HOXA11 in AML A) AML patient samples microarray datasets GSE8970 and GSE14468 were downloaded from NCBI. The correlation between UBE2E1 expression and HOXA11 gene expression was analyzed by linear regression. B) The same correlation in validation cohort was analyzed and plotted.
Supplementary Figure 3. Co-expression of UBE2E1 and HOXA11 in AML

## Slide 5
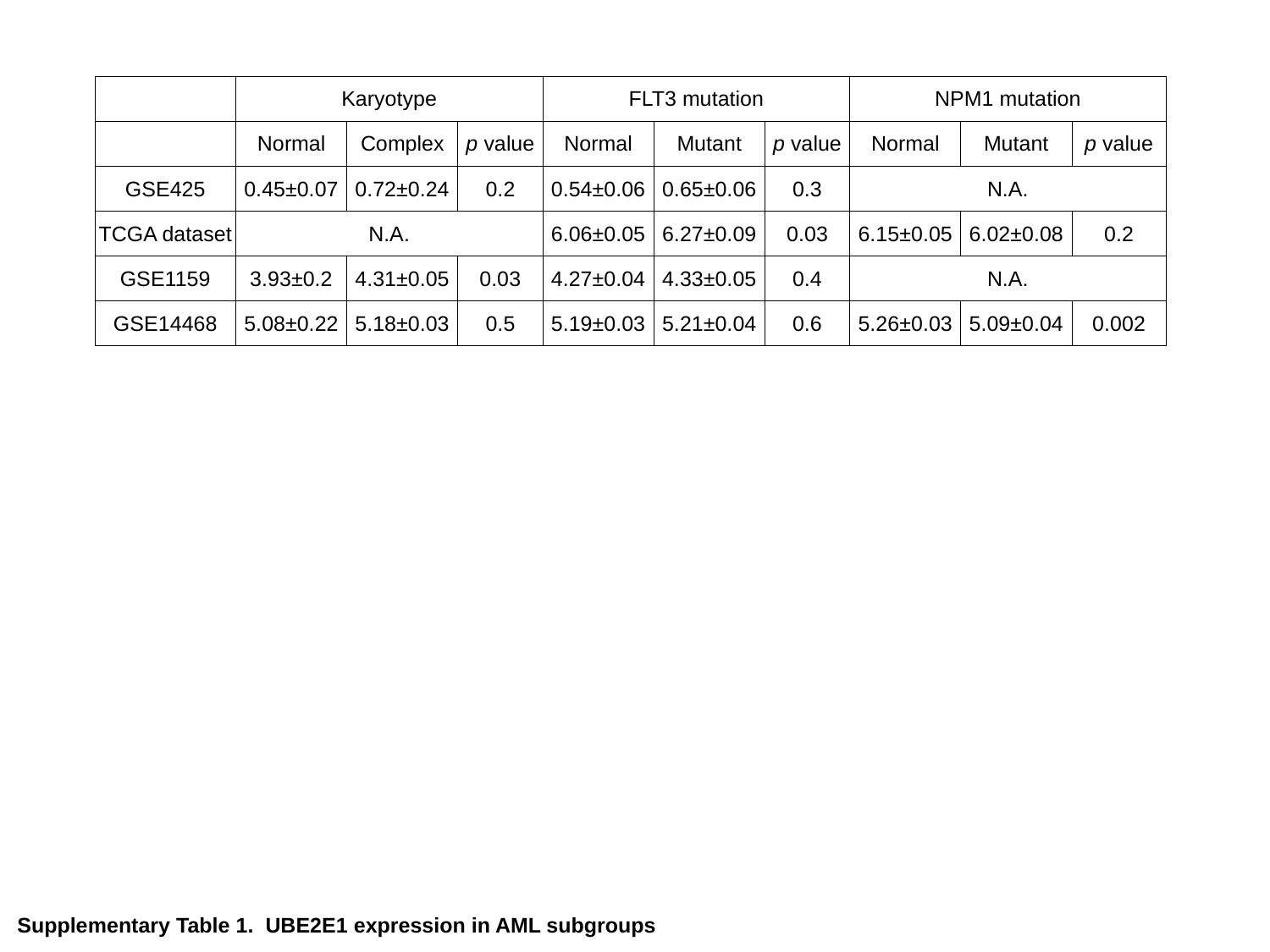

| | Karyotype | | | FLT3 mutation | | | NPM1 mutation | | |
| --- | --- | --- | --- | --- | --- | --- | --- | --- | --- |
| | Normal | Complex | p value | Normal | Mutant | p value | Normal | Mutant | p value |
| GSE425 | 0.45±0.07 | 0.72±0.24 | 0.2 | 0.54±0.06 | 0.65±0.06 | 0.3 | N.A. | | |
| TCGA dataset | N.A. | | | 6.06±0.05 | 6.27±0.09 | 0.03 | 6.15±0.05 | 6.02±0.08 | 0.2 |
| GSE1159 | 3.93±0.2 | 4.31±0.05 | 0.03 | 4.27±0.04 | 4.33±0.05 | 0.4 | N.A. | | |
| GSE14468 | 5.08±0.22 | 5.18±0.03 | 0.5 | 5.19±0.03 | 5.21±0.04 | 0.6 | 5.26±0.03 | 5.09±0.04 | 0.002 |
Supplementary Table 1. UBE2E1 expression in AML subgroups
